# Supplementary material for: SciRide Finder: a citation-based paradigm in biomedical literature search
Source: Sci Rep. 2018 Apr 18;8:6193. doi: 10.1038/s41598-018-24571-0 (PMC5906646; doi:10.1038/s41598-018-24571-0)
Supplement: Supplementary file 1 — Supplementary Information [file 41598_2018_24571_MOESM1_ESM.pdf]

SciRide Finder : a citation-based paradigm in biomedical literature search

Adam Volanakis[1], Konrad Krawczyk [1*]

1 SciRide.org

* Corresponding Author

1. Cited Statements are on average different from text content of documents they cite.

We have also measured the corresponding average difference between titles/abstracts, full text of documents and Cited Statements referring to them. For each of the 691,354 documents we have measured the average difference between Cited Statements referring to it and its title/abstract and full text (Supplementary Figure 1). For 73% of all documents, the average difference between document’s Cited Statements and title/abstract is greater than 50%. For 61% of all documents, the average difference between document’s Cited Statements and full text is at greater than 25%.

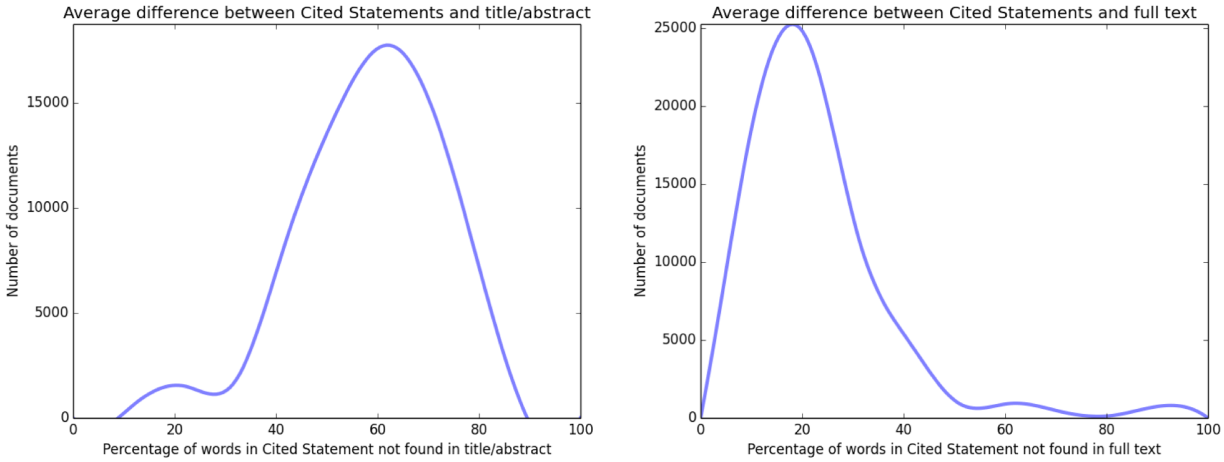

Supplementary Figure 1. Average difference between Cited Statements and text of documents they refer to. For each of 691,354 documents, we calculate the average difference between Cited Statements and title/abstract (left) and full text (right) of documents they refer to. This demonstrates that on average, Cited Statements contain significant portions of text different from the title/abstract and full text of the document they refer to.

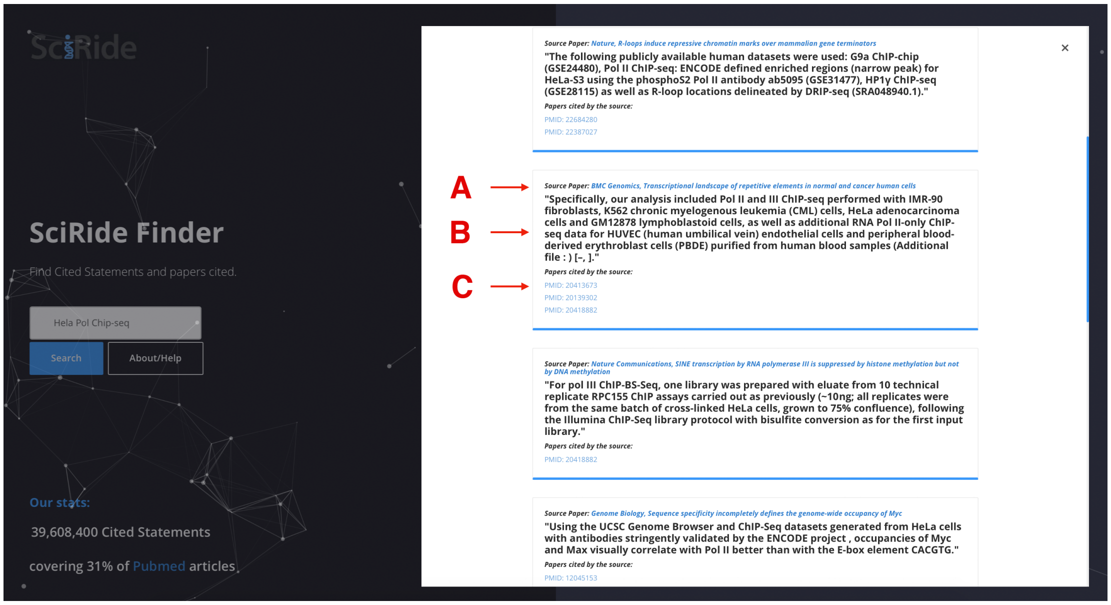

Supplementary Figure 2. Example of CS search for the identification of datasets. A search for HeLa (cell type) Pol (protein) ChIP-Seq (Method), identifies publications that have used already published datasets and provides links to the original publications where these datasets were first used. **A.** Title of the publication where the CS appears. **B.** Context where the CS appears **C.** references for the CS.

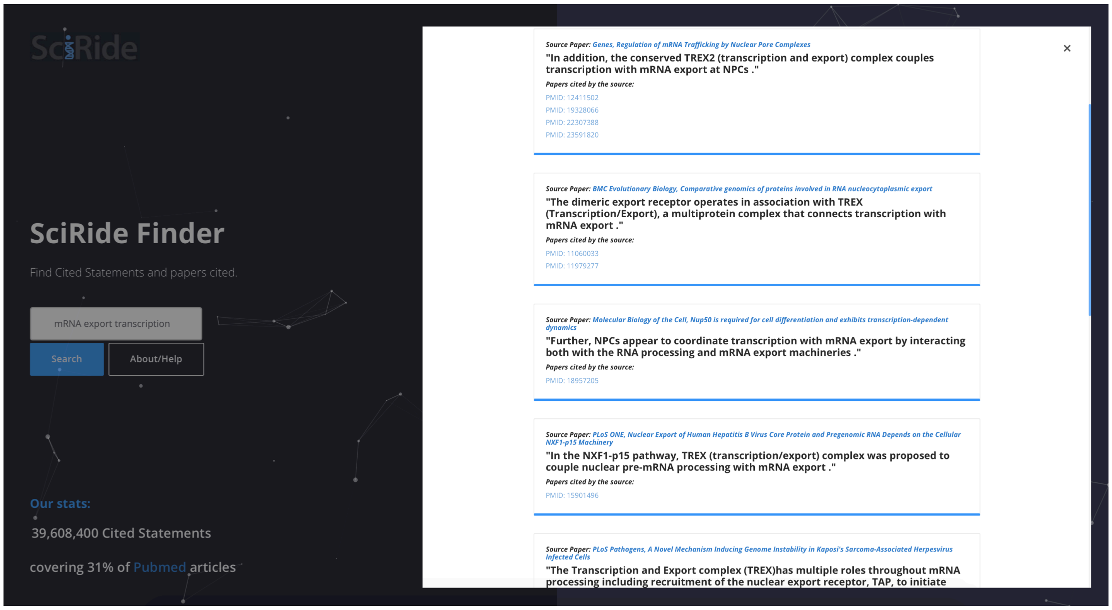

Supplementary Figure 3. Example of CS search for the appearance of two scientific terms. A search for mRNA export and transcription, returns papers identifying the overlap and coupling of the two terms as well as the original sources where these connections are based.

2. SciRide Finder as an orthogonal search engine Examples.

SciRide Finder presents results as Cited Statements – short statements from the literature supported by evidence rather than titles, abstracts or excerpts of full text. As such it differs from the established search engines in terms of retrieval strategy as well as presentation of results. Because of its nature however, it is designed to help in a set of specific scenarios which are given in comparative examples below:

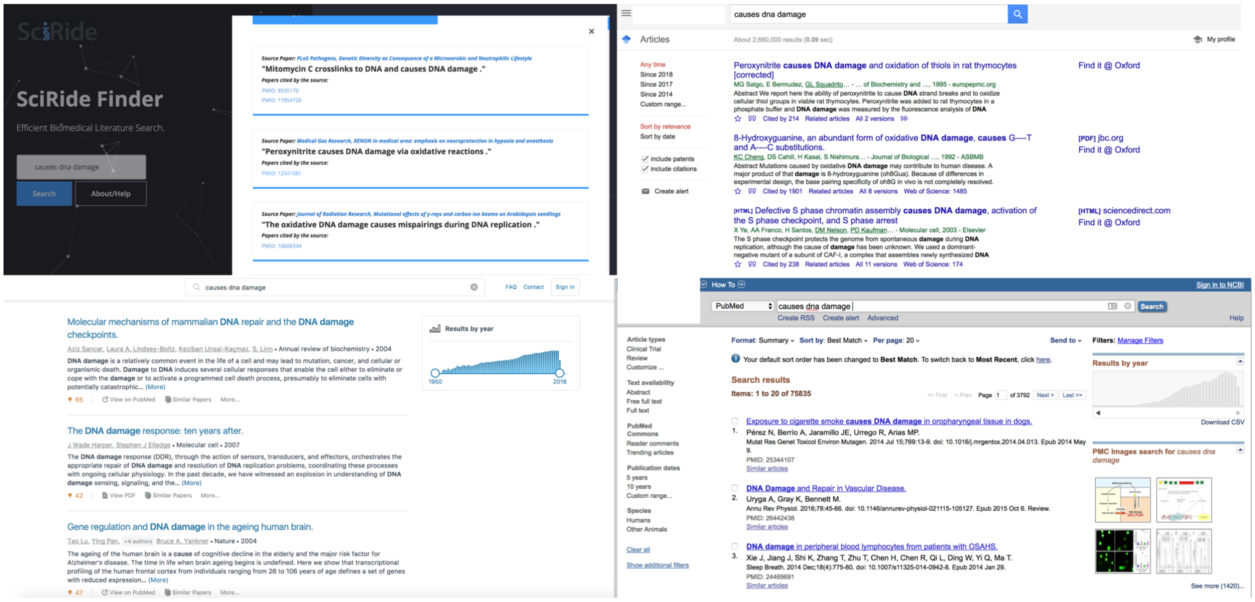

Example 1. Search scenario: DNA damage is a widely studied subject in molecular biology. Molecules/processes causing DNA damage are being commented on in the literature and can be sought after using search phrase ‘causes DNA damage’. SciRide Finder immediately shows such comments giving the user an idea what publication it is worth reading and what molecules are currently known to cause DNA damage. Other search engines however either show reviews, tasking the researcher in reading through large volumes of information, or even confuse the use of the word ‘cause’ in this case.

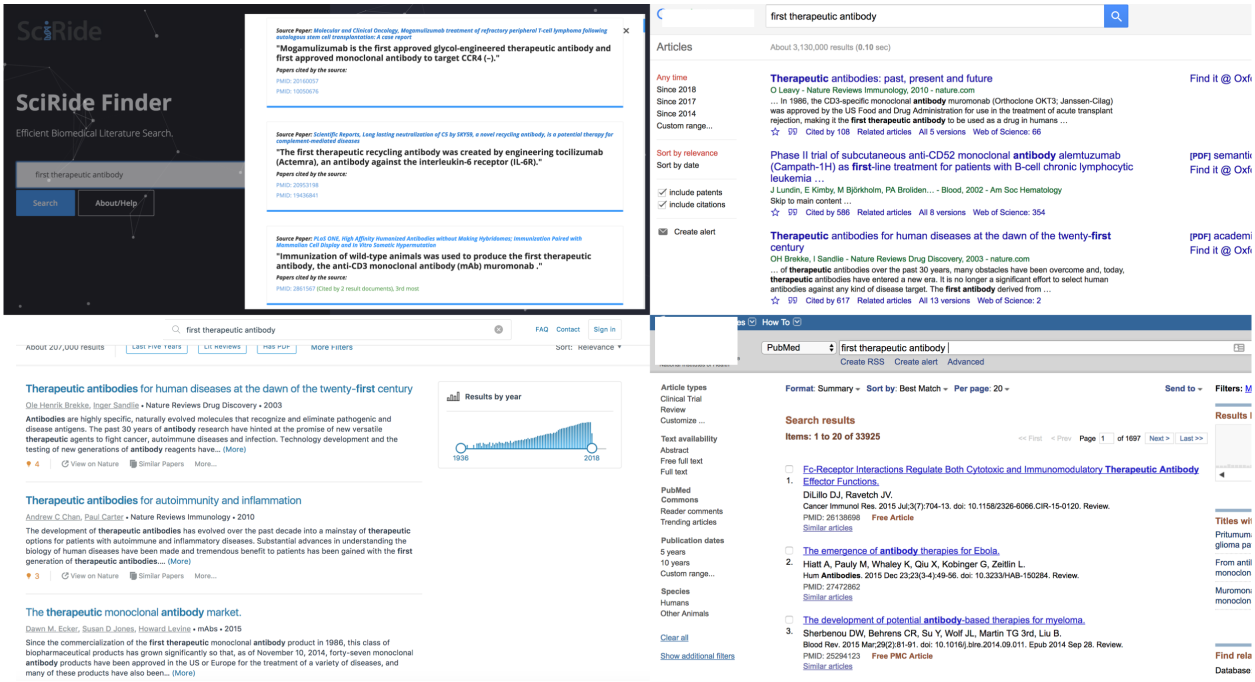

Example 2. Search scenario: find out which was the first therapeutic antibody. Results for the search phrase ‘first therapeutic antibody’ are shown for SciRide Finder, Google Scholar, Semantic Scholar and PubMed. Only SciRide Finder and Semantic Scholar correctly identify Muromab which indeed was the first therapeutic antibody.

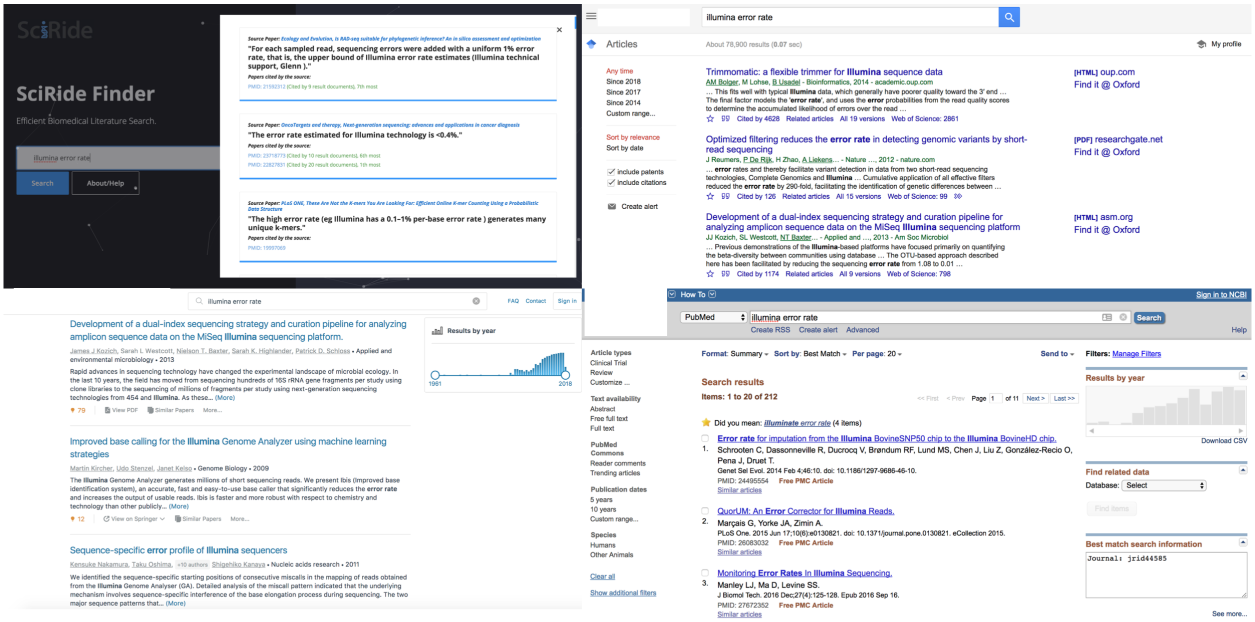

Example 3. Search scenario: find out what is the error rate of the popular Illumina sequencing platform. Search phrase used in this scenario is simply ‘illumina error rate’. SciRide finder outputs the error rate reported for the popular sequencing technology as reported in the literature. Other search engines however, output papers about the error rate of the technology. This shows the orthogonality of the approach of SciRide Finder with respect to other search engines.

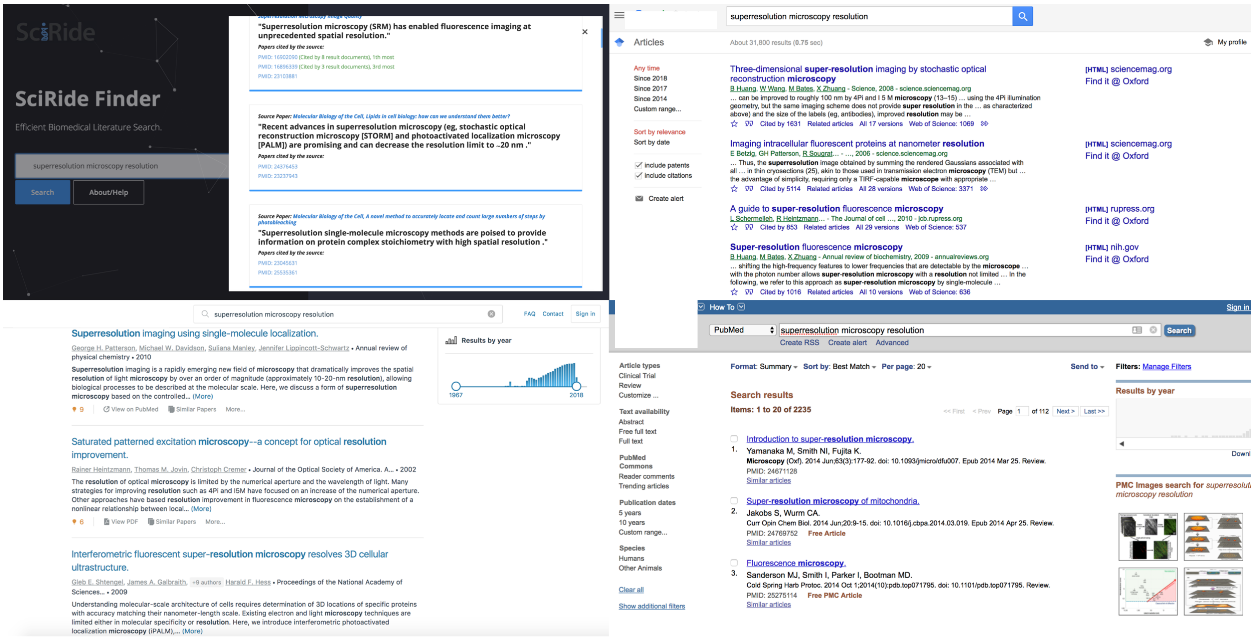

Example 4. Search scenario: superresolution microscopy is re-defining visualization of subcellular structures by achieving resolutions below the physically constraining light diffraction limit. We would like to find what is the achievable resolution by search phrase 'superresolution microscopy resolution'. Only SciRide Finder and Semantic Scholar identify the currently achieved value (e.g. achieved by Oxford Nanoimaging) of 20nm resolution.

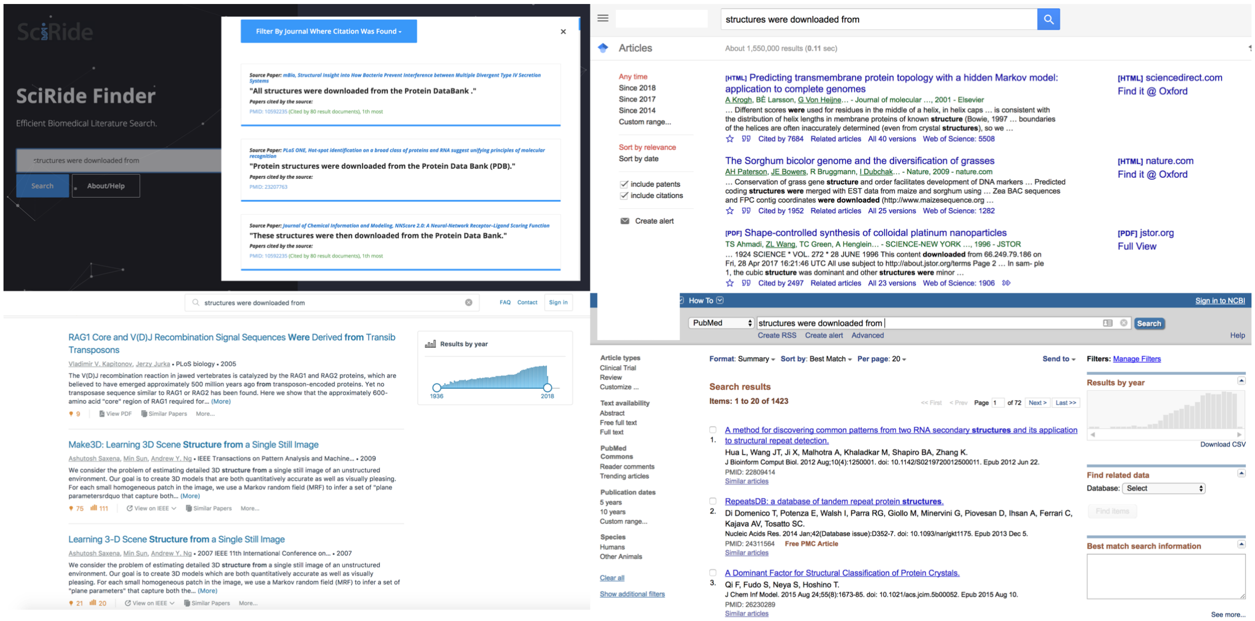

Example 5. Search scenario: Three dimensional molecular structures define the functions of proteins and studying them is facilitated via the Protein Data Bank (PDB) which is a global repository of protein structures. As a basic example of searching for data in the literature we demonstrate a scenario where someone would like to find out ‘where does one download structures from’ – which is not a trivial question for non-specialists. Searching for phrase ‘structures were downloaded from’. SciRide Finder immediately identifies phrases pointing to the PDB. Google Scholar and Semantic Scholar identify non-protein structure papers from non-medical literature because of their wide scope. Results on PubMed do not offer immediate actionable insight from the results without reading the papers returned and it is not clear whether the information would be contained in these publications. Furthermore, the word ‘downloaded’ is not typically present in the immediate results from other search engines.

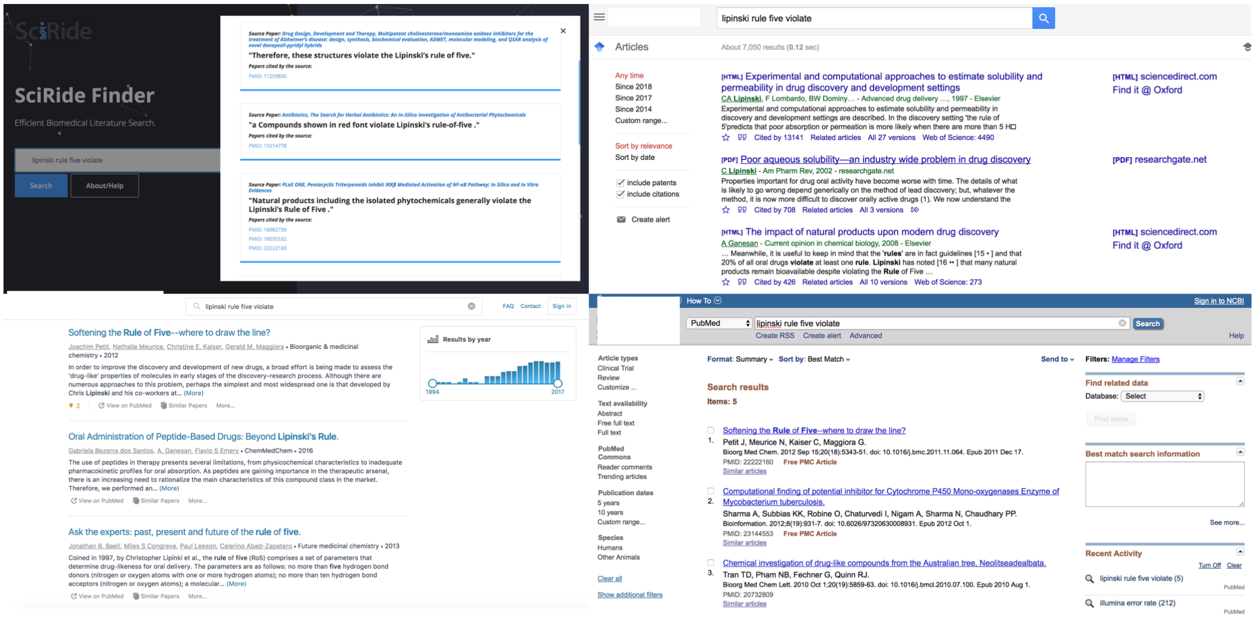

Example 6. Search scenario: Lipinski Rule of Five is a standard way to assess viability of pharmaceuticals. Developing an understanding of the implications of violation of these rules is important for pharmaceutical applications. We use search phrase ‘lipinski rule five violated’ to demonstrate the results from several search engines. SciRide Finder identifies examples where certain molecules violate the said rules, giving a researcher strong indication towards reading further to find out what are the implications of such violation. Other search engines however return publications on the Lipinski Rule of Five, do not show the word ‘violate’ in many of the presented results, offering limited clues as to which paper can answer the question.

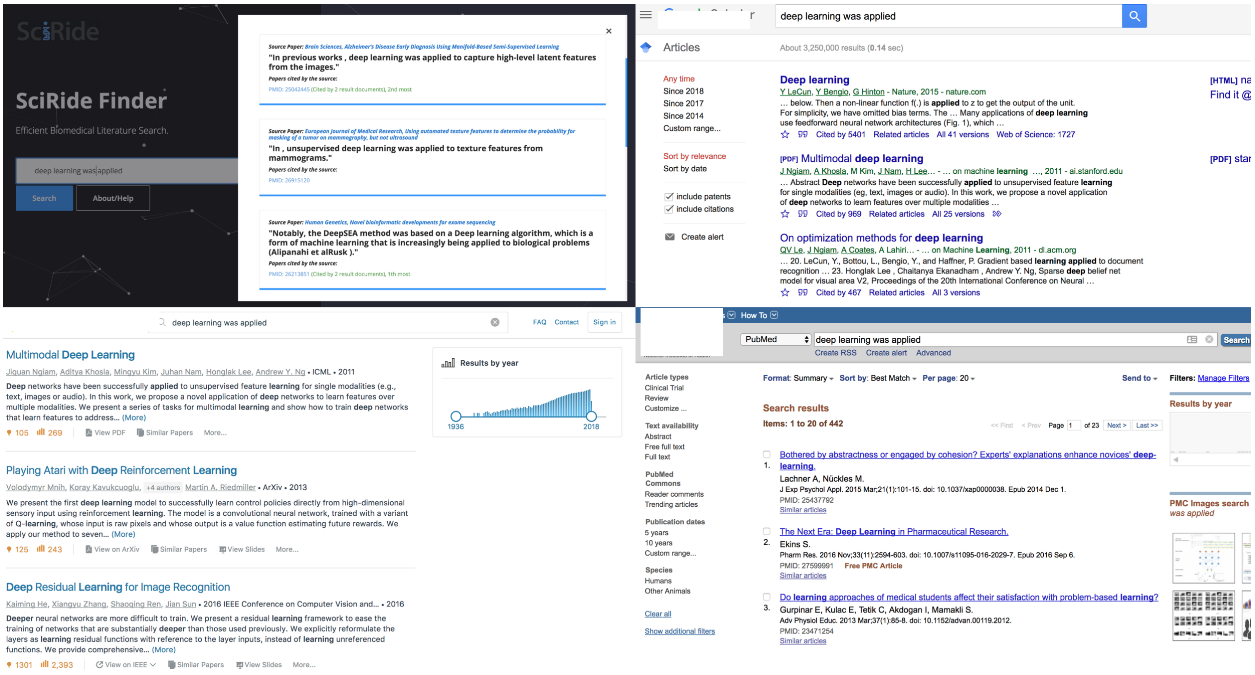

Example 7. Search Scenario: see how Deep Learning (new revolutionary machine learning paradigm) is being applied. Search phrase used here is 'deep learning was applied'. Because of the wide scope, Google Scholar and Semantic Scholar return important publications in Deep Learning in general. SciRide Finder gives indication how Deep Learning is being used in image processing and where more information can be found. PubMed appears to associate 'learning' with psychological studies.
